# DEGRO statement on the FAST-Forward trial: 10-year data of ultrahypofractionated radiation therapy for breast cancer

**DOI:** 10.1007/s00066-025-02481-1

**Published:** 2025-10-30

**Authors:** C. Matuschek, K. Borm, J. Hörner-Rieber, K. Dellas, N. M. Duma, J. Dunst, R. Fietkau, T. Hehr, M. Pazos, V. Strnad, W. Budach, M. D. Piroth, D. Krug, Breast Cancer Expert Panel of the German Society of Radiation Oncology DEGRO

**Affiliations:** 1 Medical School and University Medical Center OWL, Klinikum Mitte, Department of Radiation Oncology, Bielefeld University, Bielefeld, Germany; 2 TUM School of Medicine, Department of Radiation Oncology, Technical University of Munich, Munich, Germany; 3 Department of Radiation Oncology, University Hospital Düsseldorf, Düsseldorf, Germany; 4 Department of Radiation Oncology, University Hospital Schleswig-Holstein and Christian-Albrechts-University Kiel, Kiel, Germany; 5 Department of Radiation Oncology, Helios Clinics of Schwerin-University Campus of MSH Medical School Hamburg, Schwerin, Germany; 6 Department for Human Medicine, MSH Medical School Hamburg, Hamburg, Germany; 7 Department of Radiotherapy and Radiation Oncology, University Hospital Erlangen, Friedrich-Alexander-Universität Erlangen-Nürnberg, Erlangen, Germany; 8 Department of Radiotherapy and Radiation Oncology, Marienhospital, Stuttgart, Germany; 9 Department of Radiation Oncology, University Hospital, LMU Munich, Munich, Germany; 10 Department of Radiation Oncology, HELIOS University Hospital Wuppertal, Witten/Herdecke University, Wuppertal, Germany; 11 Department of Radiotherapy and Radiation Oncology, University Hospital Hamburg-Eppendorf (UKE), Hamburg, Germany

At the ESTRO Congress 2025, the 10-year results of the FAST-Forward trial were presented [1]. Although the full publication of the data is still pending, these long-term findings merit careful contextualization given the ongoing controversies surrounding ultrahypofractionated radiotherapy and the potential impact on clinical practice.

## Study overview

The FAST-Forward trial is a prospective randomized trial examining various fractionation schedules for adjuvant radiation therapy of breast cancer [2]. A total of 4096 patients with early-stage breast cancer (stage pT1-T3, pN0‑1, M0) treated with breast-conserving surgery (> 93%) or mastectomy (< 7%) were included. Primary endpoint was ipsilateral breast tumor relapse (IBTR). Secondary endpoints included late adverse effects, patient-reported outcome measures (PROMS), clinician-reported normal tissue effects, quality of life, breast cancer-free interval, and overall survival. In addition to the established moderately hypofractionated regimen with 40 Gy in 15 fractions over 3 weeks, two ultrahypofractionated regimens over 1 week were investigated: 26 Gy in 5 fractions of 5.2 Gy, and 27 Gy in 5 fractions of 5.4 Gy. However, the 27-Gy arm demonstrated inferior results regarding breast-specific symptoms (as reported by PROMS and physician assessment) at 5 years and will not be discussed further.

## Results

### Oncologic outcome

The estimated 10-year cumulative IBTR rate was 3.6% (95% confidence interval [CI] 2.7–4.8) in the 40-Gy arm and 2.0% (95% CI 1.3–2.9) in the 26-Gy arm [1]. The hazard ratio for the comparison of the 26-Gy arm to the 40-Gy arm was 0.62 (95% CI 0.39–0.99). No formal statistical comparison was provided since the primary endpoint analysis was performed at the 5‑year landmark.

Breast cancer free-interval and overall survival were comparable between treatment arms.

### Normal tissue effects assessed by patients and clinicians

Patients as well as clinicians documented similar adverse events in the 40- and 26-Gy arms. Absolute differences between the 40- and 26-Gy arms were mostly in the range of 0–1%.

Regarding the physician-reported breast and chest wall symptoms, which included breast induration, breast distortion, telangiectasia, breast/chest wall oedema, and breast shrinkage, moderate or marked effects were observed in 13% of patients in the 40-Gy arm, and in 14% of patients in the 26-Gy arm.

In terms of patient-reported symptoms, there were no apparent clinically relevant differences in terms of moderate and marked adverse effects for change in breast appearance (37% vs. 38%), breast shrinkage (34% vs. 35%), breast swelling (2% vs. 3%), or breast hardness/firmness (16% vs. 19%).

There were some statistically significant differences between the treatment arms at 5 years [2]. This included the prevalence of breast induration outside the tumor bed and breast or chest wall edema. The absolute differences at 5 years were minor (1.6% vs. 0.8% and 2.4% vs. 1.5%, respectively), and their clinical relevance debatable. In the presented 10-year data [1], the rates of these adverse events were comparable between the two arms, although no testing for statistical significance was provided. There was no suggestion of an increasing rate of adverse events in the 26-Gy arm with longer follow-up, which is reassuring.

Symptomatic rib fracture on the ipsilateral side (0.6% in the 40-Gy arm vs. 0.7% in the 26-Gy arm), symptomatic pulmonary fibrosis on the ipsilateral side (0.3% in the 40-Gy arm vs. 0.5% in the 26-Gy arm), and ischemic heart disease with left-sided irradiation (0.6% in both arms) were comparable.

## Limitations

The patient cohort in the FAST-Forward trial is relatively favorable in terms of risk profile, which may in part be explained by the exclusion of patients with T4 and/or N2‑3 disease. Actually, the trial was amended during enrolment to increase the primary event rate and, consequentially, stopped recruitment for low-risk patients (age ≥ 65 years, pT1, grade 1 or 2, ER positive, HER2 negative, pN0) [2].

When considering the comparison between 40 and 26 Gy in the original publication, the median age was 61 years, only 2% of patients were younger than 40 years, and 14% of patients were between 40 and 49 years old. The majority presented with early-stage disease (68% pT1, 81% pN0) and hormone receptor–positive/HER2-negative tumors (81%), while approximately 10% had HER2-positive and 9% had ER-negative/HER2-negative tumors. Chemotherapy was given to 29% of patients, mostly in the adjuvant setting.

Importantly, due to the low event rate, the trial was not sufficiently powered to evaluate recurrence rates within subgroups, thus leaving residual uncertainty about the adequacy of the regimen with 5 × 5.2 Gy for higher-risk populations. This particularly includes younger women, and those with triple-negative or HER2-positive tumors. Postmastectomy radiotherapy was delivered in only 175 patients, while breast-conserving surgery with oncoplastic techniques was used in 84 patients. All patients had pathological nodal assessment, which excludes patients in whom sentinel lymph node biopsy (SLNB) was omitted according to the INSEMA and SOUND trials [3, 4]. 

Regarding toxicity, the European Society for Radiotherapy and Oncology (ESTRO) presentation understandably lacked some details. Although the provided absolute numbers are reassuring, some ambiguity remains. The 5‑year publication reported a 19-fold increased risk of breast induration outside the tumor bed in the ultrahypofractionated arm, whereas the prevalence after 10 years for only 2345 analyzed patients was the same in both groups (1% in each group). Careful examination of the full 10-year toxicity data—which encompasses about 20 items—is warranted to clarify these findings. This includes subgroup analyses. For example, published data from FAST-Forward suggest a significantly increased risk of moderate or marked adverse events for patients < 50 years [5]. So far, there is no reason to suspect inferior outcomes with ultrahypofractionation in patients with a large breast volume [5].

It is important to notice that neither a cosmetic nor an oncologic benefit compared to moderate hypofractionation as the standard regimen has been demonstrated. Thus, the principal advantage of ultrahypofractionation lies in the shorter overall treatment duration with increased patient comfort and better utilization of health care resources as well as decreased acute skin toxicity.

This is different from what was found for moderate hypofractionation, which showed improved oncologic outcomes [6, 7] and toxicity [8] in selected trials and endpoints compared to conventional fractionation.

The advantage in terms of overall treatment time may be mitigated if an additional boost is required. While ample data are available on delivery of a simultaneous integrated boost with moderate hypofractionation [9–11], large trials studying its use with ultrahypofractionation are still ongoing, and boost irradiation was delivered sequentially with 5–8 × 2 Gy in FAST-Forward. Therefore, careful consideration of the benefits of a shorter treatment duration against the potential uncertainties is warranted.

In the 27-Gy arm of the FAST-Forward trial, a seemingly modest absolute dose escalation of 1 Gy or 3.8%—corresponding to an additional 2.8 Gy equivalent dose at 2 Gy per fraction assuming an α/β value of 2.8 for late toxicity—resulted in increases in normal tissue toxicity, as reported by both clinicians and patients. This finding highlights the steep dose–response relationship associated with ultrahypofractionated regimens, where even small variations in total dose seem to lead to clinically relevant differences in normal tissue effects.

## Recommendations of the DEGRO Expert Group for Breast Cancer

The results of the FAST-Forward trial are convincing regarding the oncologic safety of ultrahypofractionation in a predominantly low-risk patient collective. Even after 10 years, the IBTR rate is low, and at least comparable to the established moderate hypofractionation. Uncertainty remains about its use in patients with a higher overall recurrence risk, and its applicability to patients without sentinel lymph node assessment.

The recently presented 10-year data indicate that there is no significant difference regarding late toxicity, and normal tissue effects between ultrahypofractionated, and moderately hypofractionated therapy. However, it should be noted that given the limited data presented at the ESTRO annual meeting in 2025, uncertainties remain.

The evidence supporting moderate hypofractionation is extensive and consistent. Multiple randomized trials have demonstrated favorable toxicity profiles across several commonly used regimens of 40–43.5 Gy in 15–16 fractions without compromising oncologic outcomes. Collectively, these data reinforce moderate hypofractionation as the established standard of care and highlight the need for further studies on ultrahypofractionation, and careful consideration of the limitations described above.

This leads to the following assessment:

- Moderate hypofractionation with 40 Gy in 15 fractions remains standard for the majority of patients, especially those with an indication for boost irradiation.
- Ultrahypofractionation with 26 Gy in 5 fractions should be considered in patients at a low risk of recurrence with special consideration of
  - elderly and/or frail patients who are not suited to radiotherapy omission;
  - logistical reasons (e.g., long journey, pandemics, capacity limitations);
  - individual patient preferences, personal reasons, and life circumstances.
- Ultrahypofractionation should not be applied to patients with an indication for regional nodal irradiation until publication of the efficacy outcomes of the FAST-Forward nodal substudy or to patients with breast reconstruction after mastectomy.

## Conclusion

The 10-year data of the FAST-Forward trial presented at the ESTRO Congress 2025 confirm the oncologic efficacy of ultrahypofractionated radiation therapy. Regarding late toxicity and normal tissue effects, outcomes were similar for 26 Gy in 5 fractions vs. 40 Gy in 15 fractions. It represents a useful addition to the radiotherapeutic arsenal, and complements treatment options for individualized and tailored treatment of patients with early breast cancer. Final evaluation is, however, reserved for the upcoming full publication.
